# Supplementary material for: Causal Relationship between Aging and Anorexia Nervosa: A White-Matter-Microstructure-Mediated Mendelian Randomization Analysis
Source: Biomedicines. 2024 Aug 16;12(8):1874. doi: 10.3390/biomedicines12081874 (PMC11351342; doi:10.3390/biomedicines12081874)
Supplement: Supplementary file 1 [file biomedicines-12-01874-s001.zip › Supplementary Figure/Figure S4/Singe SNP plot of MR analysis for TL to ubm-b-1804.pdf]

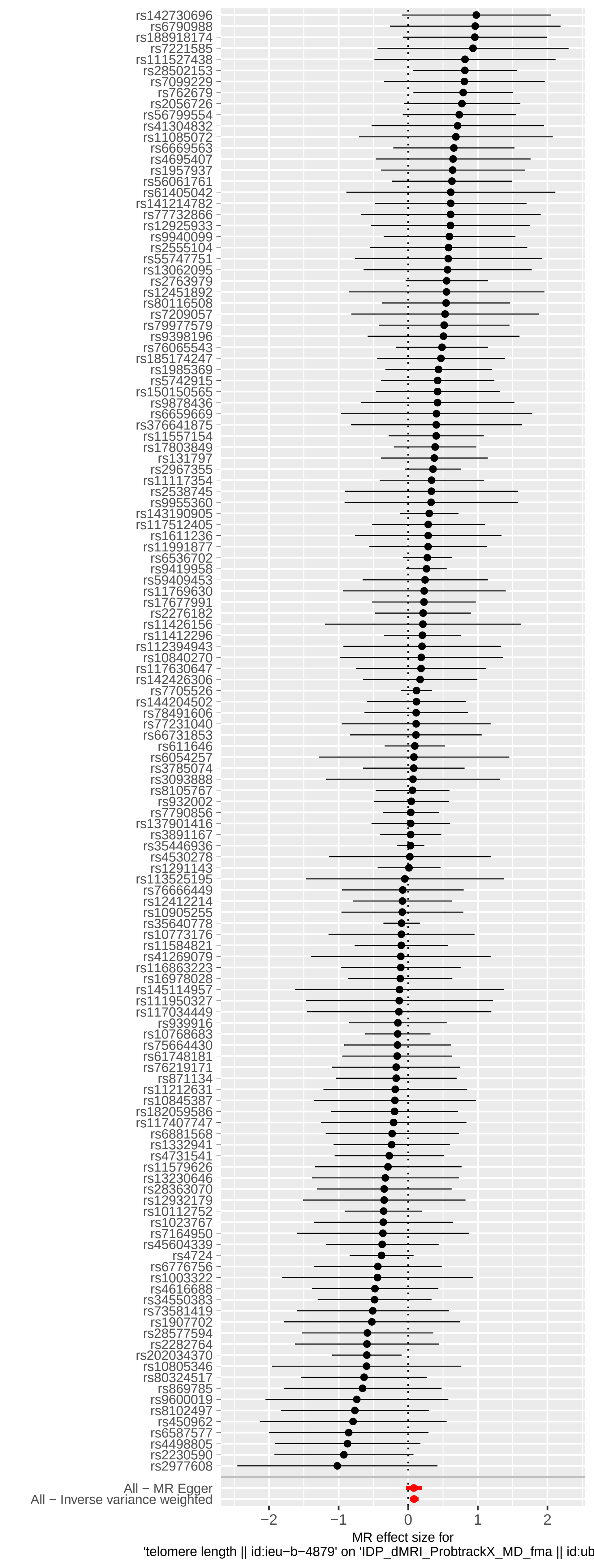
rs142730696
rs6790988
rs188918174
rs7221585
rs111527438
rs28502153
rs7099229
rs762679
rs2056726
rs56799554
rs41304832
rs11085072
rs6669563
rs4695407
rs1957937
rs56061761
rs61405042
rs141214782
rs77732866
rs12925933
rs9940099
rs2555104
rs55747751
rs13062095
rs2763979
rs12451892
rs80116508
rs7209057
rs79977579
rs9398196
rs76065543
rs185174247
rs1985369
rs5742915
rs150150565
rs9878436
rs6659669
rs376641875
rs11557154
rs17803849
rs131797
rs2967355
rs11117354
rs2538745
rs9955360
rs143190905
rs117512405
rs1611236
rs11991877
rs6536702
rs9419958
rs59409453
rs11769630
rs17677991
rs2276182
rs11426156
rs11412296
rs112394943
rs10840270
rs117630647
rs142426306
rs7705526
rs144204502
rs78491606
rs77231040
rs66731853
rs611646
rs6054257
rs3785074
rs3093888
rs8105767
rs932002
rs7790856
rs137901416
rs3891167
rs35446936
rs4530278
rs1291143
rs113525195
rs76666449
rs12412214
rs10905255
rs35640778
rs10773176
rs11584821
rs41269079
rs116863223
rs16978028
rs145114957
rs111950327
rs117034449
rs939916
rs10768683
rs75664430
rs61748181
rs76219171
rs871134
rs11212631
rs10845387
rs182059586
rs117407747
rs6881568
rs1332941
rs4731541
rs11579626
rs13230646
rs28363070
rs12932179
rs10112752
rs1023767
rs7164950
rs45604339
rs4724
rs6776756
rs1003322
rs4616688
rs34550383
rs73581419
rs1907702
rs28577594
rs2282764
rs202034370
rs10805346
rs80324517
rs869785
rs9600019
rs8102497
rs450962
rs6587577
rs4498805
rs2230590
rs2977608
All − MR Egger
All − Inverse variance weighted
−2
−1
0
1
2
MR effect size for
'telomere length || id:ieu−b−4879' on 'IDP_dMRI_ProbtrackX_MD_fma || id:ub
